# A massive pleural‐based desmoid tumour

**DOI:** 10.1002/rcr2.205

**Published:** 2016-12-01

**Authors:** Talha Mahmud, Guness Mal, Farhan Ahmed Majeed, Siaw Ming Chai, Y C Gary Lee

**Affiliations:** ^1^ Department of PulmonologyShaikh Zayed Hospital, FPGMILahorePakistan; ^2^ Department of Thoracic SurgeryCombined Military HospitalLahorePakistan; ^3^ Department of Anatomical PathologyPathWest Laboratory Medicine, QEII Medical CentrePerthAustralia; ^4^ Pleural Medicine UnitInstitute for Respiratory HealthPerthAustralia; ^5^ Department of Respiratory MedicineSir Charles Gairdner HospitalPerthAustralia; ^6^ School of Medicine and PharmacologyUniversity of Western AustraliaPerthAustralia

**Keywords:** Desmoid, effusion, mesothelial, pleura

## Abstract

A 49‐year‐old Pakistani male presented with “heaviness” in his chest. Chest radiograph and computed tomography (CT) confirmed a massive left‐sided pleural‐based opacity. Three years ago, he was investigated for a left‐sided lymphocytic, exudative pleural effusion following an episode of dengue fever. Tube thoracostomy removed 1.3 L of fluid. Pleural biopsy and bronchial washings were non‐contributory. He received empirical anti‐tuberculosis treatment and remained asymptomatic until this presentation. To investigate the new pleural mass, he underwent a video‐assisted thoracoscopic surgery, which revealed a 2.2 kg mass in the pleural cavity involving the anterior mediastinum and chest wall and adhered to the visceral pleura. Following conversion to an open thoracotomy, the mass was completely excised, which involved non‐anatomical lung resection. Histopathology and immunohistochemistry of the resected tumour were consistent for a desmoid tumour. He was followed up for 9 months with no evidence of tumour recurrence. Predominantly pleural‐based desmoid tumour is rare but should be included in the differential diagnosis of spindle cell tumours.

## Introduction

Pleural tumours and associated effusions are common clinical presentations. Most pleural tumours are malignant although benign ones (e.g. solitary fibrous tumour of the pleura (SFTP)) occasionally occur. This case describes the presentation of desmoid tumour as a rare cause of pleural tumour.

## Case Report

A 45‐year‐old businessman was hospitalized for 5 days for dengue fever in 2011 during the peak of a dengue epidemic in Pakistan. He was managed with supportive treatment. Other than controlled hypertension, his past medical and family histories were unremarkable. A week post‐discharge, he re‐presented with left‐sided chest pain, exertional dyspnoea, cough and a medium‐sized, multi‐loculated left‐sided pleural effusion. Baseline haematological, coagulation and biochemical profiles were normal. A diagnostic pleural aspiration revealed a straw‐coloured lymphocyte‐predominant exudate. Bronchoscopy and bronchial washings were non‐informative. A closed Abrams’ biopsy yielded only non‐specific pleural inflammation. A chest tube was inserted and 1.3 L of fluid was removed. In accordance with local practice for undiagnosed lymphocytic effusions, he was given an empirical trial of quadruple anti‐tuberculosis (TB) therapy for TB pleuritis which he took for 4 months before being lost to follow‐up.

**Figure 1:**
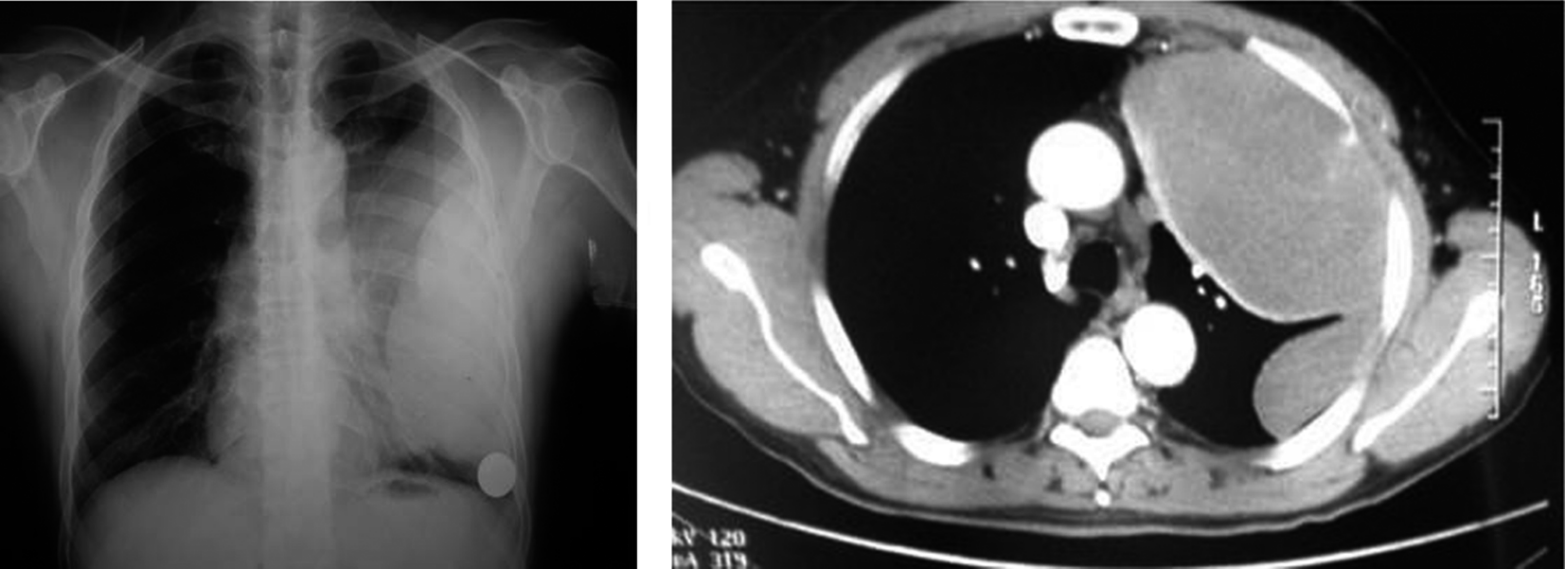
(A) Chest radiograph and (B) computed tomography at the time of presentation in 2015 showing a pleural‐based opacity involving middle and lower zones of left hemithorax.

**Figure 2:**
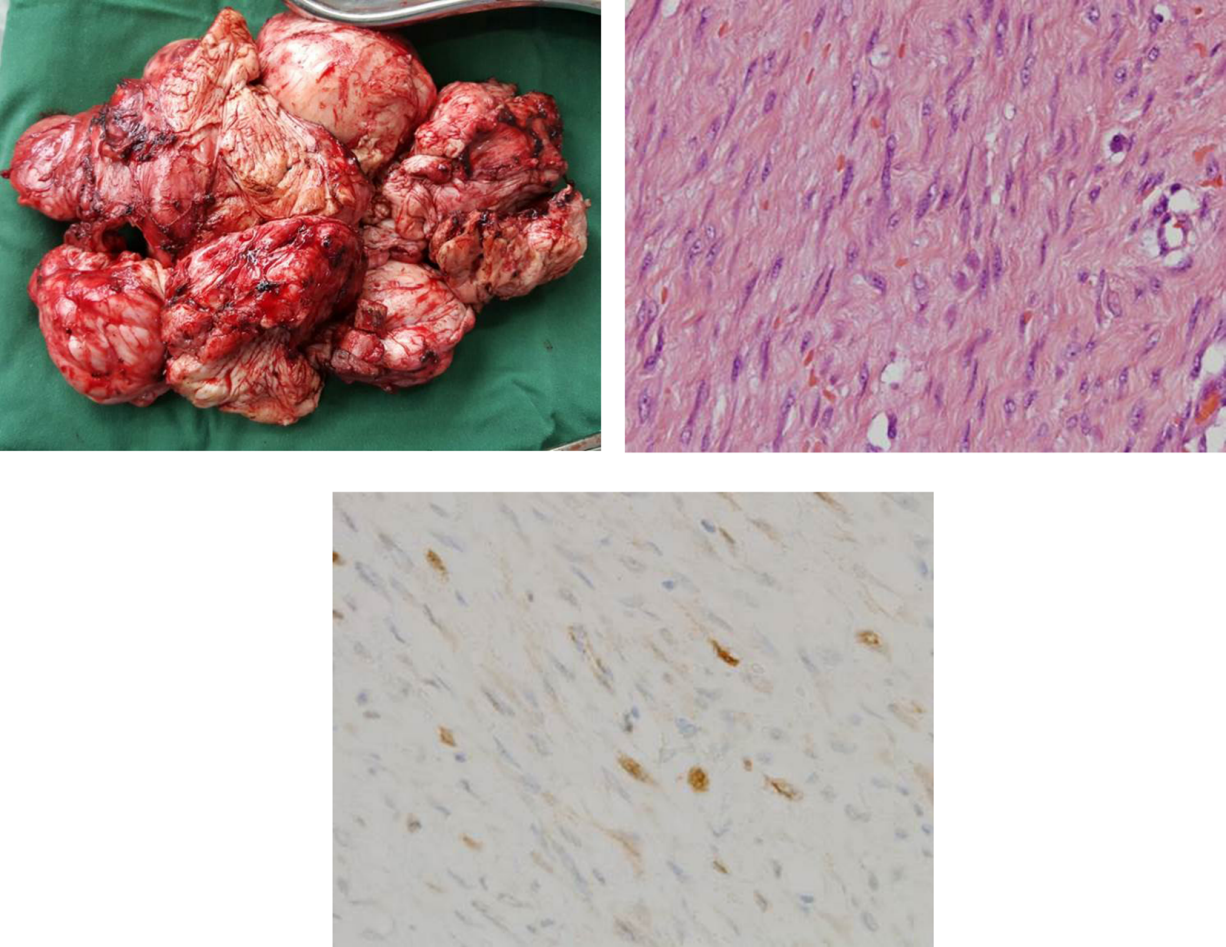
(A) The excised tumour weighed 2.2 kg. (B) Histologically, it was characterized by bland spindle‐shaped cells arranged in fascicles (haematoxylin–eosin, original magnification ×200) with (C) focal nuclear β‐catenin positivity (original magnification ×200).

He re‐presented in June 2015 with “heaviness” in his chest. A chest X‐ray (CXR) and computed tomography (CT; Fig. 1) showed a large left‐sided pleural‐based low‐density mass resembling a pleural effusion. The previous loculated residual pleural fluid collections had resolved. There was visceral pleural thickening but no abnormality was seen in the lung parenchyma or cardiovascular structures. He underwent a video‐assisted thoracoscopic surgery (VATS), which identified a huge, firm‐to‐hard, pleural‐based mass that was completely excised following conversion to a thoracotomy. The mass (Fig. 2A), which weighed 2.2 kg, occupied the anterior mediastinal recess at the level of left internal mammary artery and involved the second and third intercostal recessions. The mass was parietal pleural‐based but also extended into the chest wall and was adherent to the visceral pleura, thus requiring non‐anatomical lung resection for removal. The histopathology was unusual. An opinion was sought from the multi‐disciplinary thoracic oncology meeting of a quaternary centre of pleural disease/mesothelioma. Tissue block and slides were examined by a specialist pleural pathologist (S. M. Chai).

The morphology and immunohistochemistry of the tumour were diagnostic for desmoid‐type fibromatosis. The lesional tissue showed bland spindle‐shaped cells arranged in intersecting fascicles (Fig. 2B). The cells were set in variably dense collagenous stroma, with patches of myxoid change. The spindle‐shaped cells had oval nuclei, small nucleoli and delicate pale eosinophilic to amphophilic cytoplasm. Scattered mitotic figures were present without any necrosis or cellular atypia. At the periphery of the tumour, the spindle‐shaped cells infiltrated and surrounded individual skeletal muscle fibres. There was patchy cytoplasmic staining for smooth muscle actin and nuclear staining for β‐catenin (Fig. 2C). The lesional cells were negative for desmin, S‐100, calretinin, MNF 116, D2‐40, AE1 + 3, CK7 and CD34 immunostains.

The patient made an uneventful recovery and had no signs of tumour recurrence after 9 months of follow‐up.

## Discussion

Pleural tumours are often malignant, either arising from the pleura (commonly mesothelioma) or from metastatic spread of extrapleural cancer. Benign pleural tumours, for example, SFTP, are significantly less common. A desmoid tumour predominantly involving the pleural cavity has rarely been reported. 1

Desmoid tumours, or desmoid‐type aggressive fibromatoses, are clonal fibroblastic proliferations characterized by locally aggressive tumours which do not metastasize but often recur despite resection 2. Desmoid tumours occur usually between 15 and 60 years of age and have no strong gender preponderance and no ethnic predilection.

Desmoid tumours can affect any part of the body. In patients with familial adenomatous polyposis (FAP), intra‐abdominal desmoids (involving the bowel and mesentery) predominate. In a series of 234 desmoid tumours, the largest extra‐abdominal sites were in the foot, shoulder, thigh and calf 2. About one‐fifth of desmoid tumours are thoracic in location; the majority arises in the chest wall (and can be palpable), and some in the mediastinum and lung. The pleura may be secondarily involved. Rarely however had desmoid tumour been reported to predominantly involve the pleural cavity.

Most desmoid tumours arise sporadically. Genetic mutations (e.g. that of CTNNB1 gene) which activate β‐catenin are common 3. Some (5–15%) cases may be associated with FAP due to mutations in the APC gene on chromosome 5q21–q22 4. Our patient had no symptoms or family history of FAP and declined a colonoscopy.

He had an unusual presentation of a preceding lymphocytic effusion nearly 4 years before presenting with this desmoid tumour. It is unclear if the two events are causally related. However, antecedent trauma (e.g. thoracic surgery 5) – which triggers aberrant wound healing and fibroproliferative disorders of mesenchymal tissue – is a recognized aetiological factor. Whether the prior tube thoracostomy in our patient had precipitated the development of desmoid growth was unclear.

The clinical course of desmoid tumours is quite unpredictable, and recurrence occurs in up to 39% of patients even after resection with clear margins. Further surgery and/or radiotherapy are considered if local recurrence develops.

This report highlights desmoid tumour as a potential cause of pleural‐based tumours, and must be included in the differential diagnosis of spindle cell neoplasms and/or proliferations of the pleura (including sarcoma, sarcomatoid mesothelioma, SFTP, etc.).

### Disclosure Statements

No conflict of interest declared.

Appropriate written informed consent was obtained for publication of this case report and accompanying images.

### Funding Statement

Y. C. G. Lee is a National Health and Medical Research Council (NHMRC) Career Development Fellow and receives research project grant funding from the NHMRC, New South Wales Dust Disease Board, Sir Charles Gairdner Research Advisory Committee, Westcare and the Cancer Council of Western Australia.
